# Anatomical variations of the mental foramen in prognathic, retrognathic and orthognathic mandibles: a cone-beam computed tomography based analysis

**DOI:** 10.3389/fdmed.2026.1839094

**Published:** 2026-07-22

**Authors:** Supriya Bhat, Ravi Marakini Subrahmanya, Vidya Ajila, M. N. Kuttappa

**Affiliations:** 1 Department of Oral Medicine and Radiology, AB Shetty Memorial Institute of Dental Sciences (ABSMIDS), Nitte (Deemed to be University), Mangalore, India; 2 Department of Orthodontics and Dentofacial Orthopedics, AB Shetty Memorial Institute of Dental Sciences (ABSMIDS), Nitte (Deemed to be University), Mangalore, India

**Keywords:** CBCT, cephalometrics, mandible, mental foramen, skeletal malocclusion

## Abstract

**Introduction:**

The mental foramen is a key anatomical landmark that transmits the mental nerve and vessels. Its position varies across individuals and skeletal patterns, making accurate localization essential during surgical procedures such as local anesthesia, implant placement, and dental extractions. The objective of this study was to assess the horizontal position of the mental foramen, the distance from the mental foramen to the adjacent tooth apex and the distance from the superior border of the mental foramen to the inferior border of the mandible in individuals with prognathic, retrognathic, and orthognathic mandibles using cone-beam computed tomography (CBCT).

**Material and methods:**

CBCT-derived lateral cephalograms of 39 subjects were used to identify cephalometric landmarks and classify subjects into three skeletal groups. Mental foramen position was assessed using standard references, with group differences analyzed by Fisher-Freeman-Halton exact test and one-way ANOVA (*p* < 0.05).

**Results:**

The position of the mental foramen was classified from P1-P5 based on their relationship to the mandibular teeth: P1, anterior to first premolar; P2, in line with the first premolar; P3, between first and second premolar; P4, in line with the second premolar; P5, between second premolar and first molar. The predominant position in the retrognathic mandible was P5–46.2% (right) and 38.5% (left). In the prognathic mandible, P4 was the most common position—53.8% (right), while P3 (46.2%) predominated in the left. In orthognathic mandibles, P3 (69.2%) was the most common position on both sides. Significant differences were observed in the position of the mental foramen between the groups only on the right side. Significant sex differences were seen when the distance from the superior border of the mental foramen to the inferior border of the mandible was compared.

**Conclusions:**

Significant differences in the horizontal position of the mental foramen among skeletal patterns was observed on the right side. The distance between mental foramen and adjacent root apex demonstrated a significant right-left difference in the retrognathic group. CBCT may aid in the preoperative localization of the mental foramen for mandibular surgical planning.

## 1 Introduction

The mental nerve and vessels exit through a vital anatomical landmark—Mental foramen (MF) (1). The mental nerve supplies the lower lip, chin and mandibular labial gingiva (2). It is located on the buccal cortical bone of the mandible usually in the premolar region with considerable individual and population variation. Accurate determination of the same is vital for clinicians to facilitate local anesthesia to carry out procedures like incisions, flaps, endodontic surgery, dental implants, dental extraction, trauma and orthognathic surgeries. Complications associated with the same can be due to toxicity secondary to local anesthetic, ineffective anesthesia, formation of hematoma and injury to the nerve (3).

Cone beam computed tomography (CBCT) has transformed the dental scenario with its ability to generate high-resolution, three-dimensional (3D) images of the oral as well as maxillofacial structures. This technology provides an extensive view of the dentition, bone and nerve pathways, enabling accurate diagnosis and treatment planning (4).

Although the position of the mental foramen has been extensively studied using various imaging modalities and on dry mandibles, there is limited literature evaluating its relationship with sagittal skeletal patterns of the mandible (1, 3). Moreover, studies correlating mental foramen location with prognathic, retrognathic, and orthognathic mandibular morphology using CBCT are scarce. Therefore, the present study aims to evaluate the position of the mental foramen in prognathic, retrognathic, and orthognathic mandibles in an age-specific adult population (18–30 years) using CBCT, with the hypothesis that the position of the mental foramen varies according to sagittal skeletal pattern.

## 2 Materials and methods

This cross-sectional analytical study included analysis of 78 mental foramina (right and left sides) in 39 subjects from the Department of Orthodontics and Dentofacial Orthopedics at a South Indian institution. Sample size was determined using a formal sample size calculation with G*Power software considering an effect size of 0.5, Power 80%, allocation ratio 1:1, degree of freedom—2% and 5% Type 1 Error and estimated as 39 (5). The calculation was based on categorical outcome. Although cross-sectional in design, participants were prospectively recruited, and CBCT acquisition was standardized to ensure uniform measurement quality. The study was carried out in accordance with the Declaration of Helsinki and was approved by the Central Ethics Committee of the institution, with NU/CEC/2023/491. Written informed consent was procured from each individual. Ethical clearance was obtained from the Central Ethics Committee before the start of the study.

Individuals recommended for Full Volume CBCT analysis from the Department of Orthodontics and Dentofacial Orthopedics were included in the study. All CBCT examinations included in the study were a part of orthodontic treatment planning. No scans were acquired solely for research purposes. Cone- beam computed tomography images were procured using Planmeca Promax 3D CBCT unit (Planmeca Oy, Helsinki, Finland) in an upright seated position with the Frankfort horizontal plane oriented parallel to the floor and the midsagittal plane perpendicular to the floor under standardized conditions. All CBCT scan volumes included in the study were obtained using the same imaging unit with standardized protocols, featuring a 20 × 17.4 cm field of view (FOV), a voxel size of 0.4 mm, and exposure parameters set at 90 kVp, 10 mA, and 8–13 s. Multiplanar reconstruction images in axial, coronal and sagittal sections were generated using the Planmeca Romexis Viewer software 4.6.2R. The reconstructed slice thickness corresponded to the acquired voxel size of 0.4 mm. Prior to linear measurements, the CBCT volumes were reoriented in the axial, coronal, and sagittal planes using the Frankfort horizontal plane and midsagittal plane as reference planes to ensure standardized image orientation and consistent measurements.

**Figure 1:**
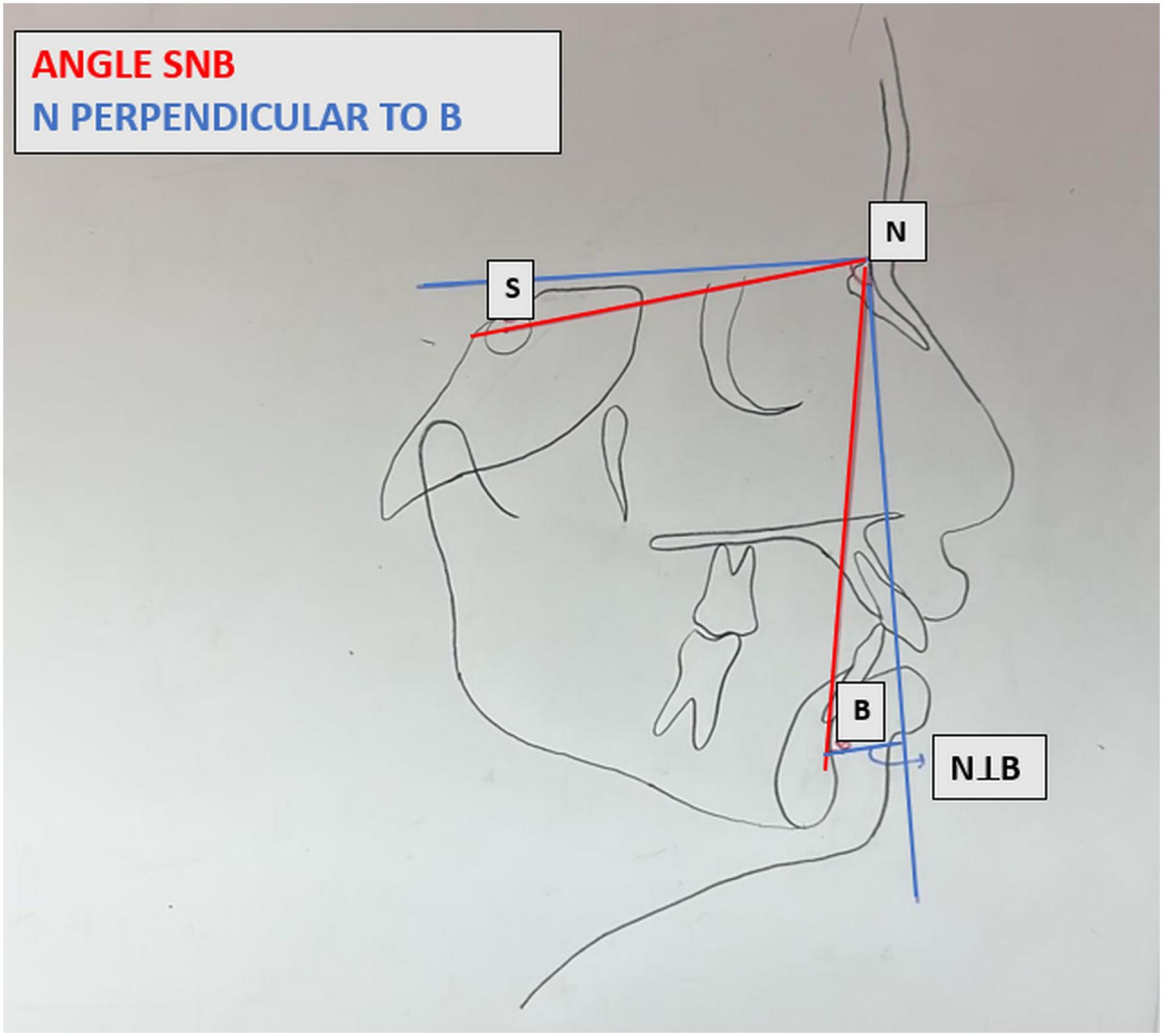
Lateral cephalometric tracing showing SNB angle and N perpendicular to point B (N perpendicular to B).

Lateral Cephalograms were generated from the CBCT scan volumes, and cephalometric landmarks were identified. Images were analyzed for mandibular morphology using SNB angle (angle between the Sella-Nasion and the Nasion-B lines) and N perpendicular to B measurement as described in established cephalometric analyses for assessment (6) (Figure 1). The subjects were grouped into Prognathic, Retrognathic and Orthognathic mandibles.

- Group 1- Prognathic Mandible (N perpendicular to B with values—Males = >1.4 mm, Females = > −2.6 mm, Angle SNB >86^0^
- Group 2- Retrognathic Mandible (N perpendicular to B with values—Males = <−12 mm, Females = <−11.2 mm, Angle SNB <76^0^)
- Group 3—Orthognathic Mandible (N perpendicular to B with values—Males =  −5.3 mm (±6.7 mm), Females = −6.9 mm (±4.3 mm), Angle SNB = 78–82^0^)

Subjects in the age group of 18–30 years with no history of facial or surgical treatment were included. Individuals with systemic diseases, periodontal and gingival diseases, facial asymmetry, craniofacial syndromes and individuals with multiple missing teeth were excluded from the study.

**Figure 2:**
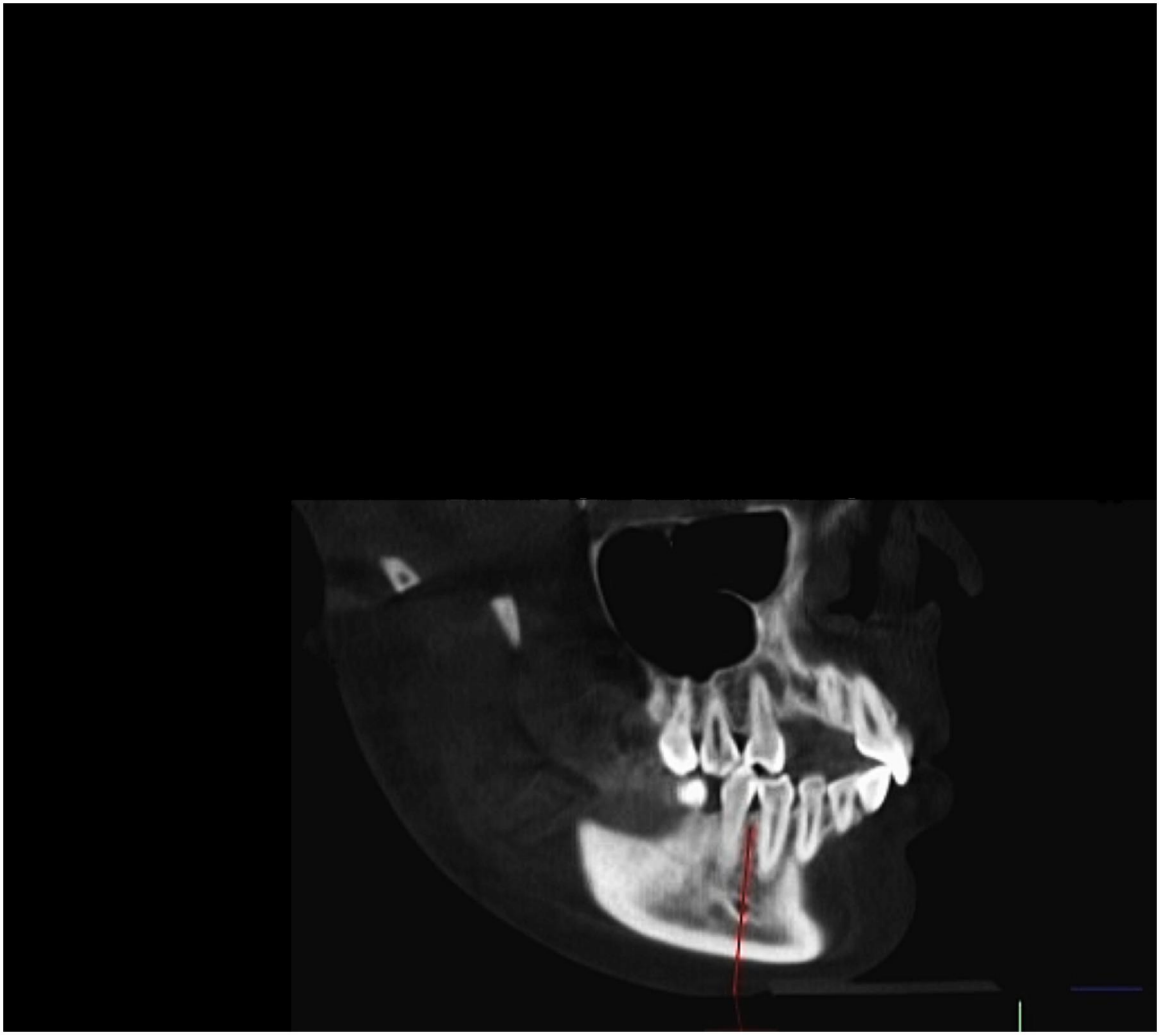
Cropped sagittal section showing the horizontal position of the mental foramen between first and second premolars (P3).

The location of the mental foramen was initially identified in the axial section. Using multiplanar reconstruction, the corresponding sagittal section was obtained. The sagittal slice showing maximum visualization of the mental foramen was selected for measurements. Horizontal position of the foramen, distance from the mental foramen and the adjacent root apex and the distance from the superior portion of the mental foramen and inferior border of the mandible were recorded. For an accurate determination of location, the vertical midline of the MF was taken into consideration as a standard reference point as follows (Figure 2). The vertical midline of the mental foramen corresponds to the line passing through the center of the foramen determined by considering the most superior, inferior, anterior and posterior corticated borders.

P1: The foramen is situated anterior to the first premolar.

P2: The foramen is situated in line with the first premolar.

P3: The foramen is situated between the first and second premolar.

P4: The foramen is situated in line with the second premolar.

P5: The foramen is situated between the second premolar and first molar (7).

**Figure 3:**
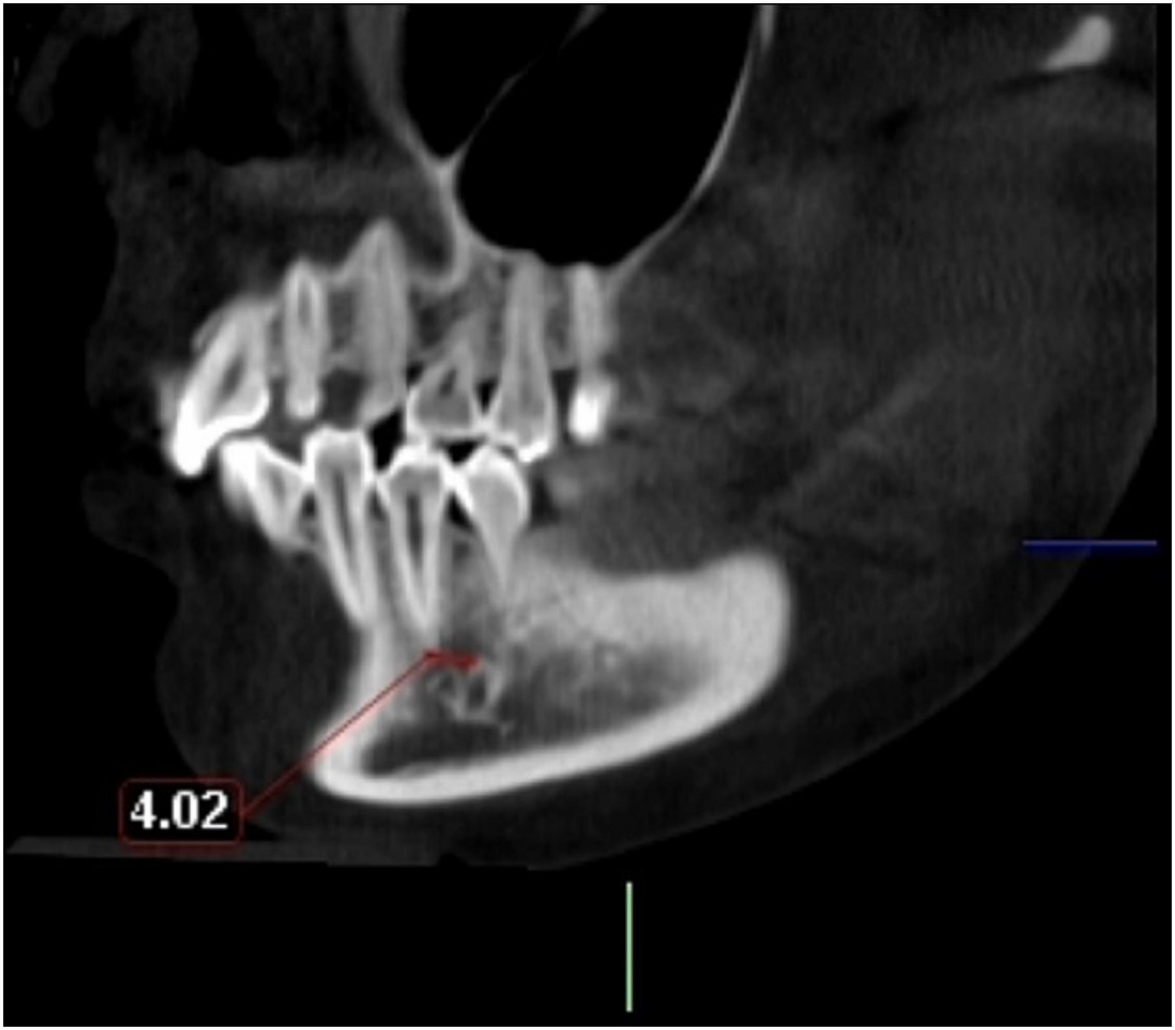
Cropped sagittal section illustrating the measurement from the adjacent root apex to the most superior continuous corticated outline of the mental foramen.

**Figure 4:**
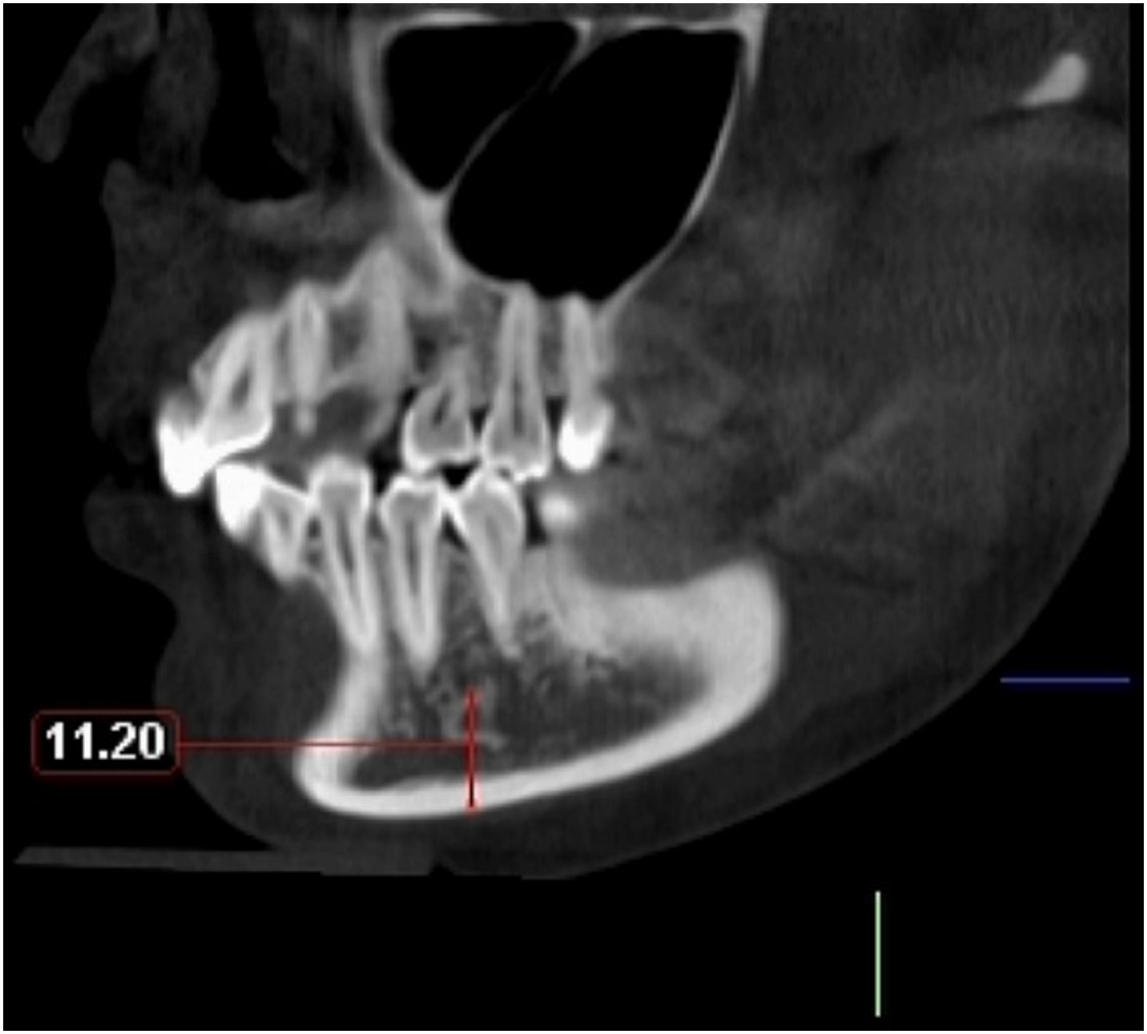
Cropped sagittal section illustrating the distance between the superior border of the mental foramen and inferior border of the mandible.

The distance from the mental foramen to the adjacent root apex was determined on sagittal sections of CBCT as the shortest distance between the superior border of the mental foramen and the adjacent root apex (Figure 3). The superior margin was identified as the most superior continuous corticated outline of the foramen. Measurements were recorded on the sagittal slice, demonstrating maximum visualization of the foramen. The shortest distance between the superior border of the mental foramen and the inferior border of the mandible was also recorded (Figure 4). The unit of measurement was millimetres (mm). Accessory mental foramina, bifid mental canals and anterior loops of the inferior alveolar nerve were not included as study variables. When additional foramina or anatomical variations were observed, they were not included for analysis as their assessment was beyond the scope of the present study.

The cephalometric analysis and classification into study groups were done by an Orthodontist. The study data were anonymized and blinded before evaluation. The CBCT-based measurements were independently done by an Oral and Maxillofacial Radiologist. To assess intra-examiner consistency, the Oral radiologist repeated the measurements after a two-week interval.

### 2.1 Statistical analysis

All data were compiled in Microsoft Excel. The analysis was done using IBM SPSS Statistics (Version 23). The Shapiro–Wilk test was done to assess the normality of the variables. Homogeneity of variances was assessed using Levene's test prior to application of one-way ANOVA, and the assumptions for parametric testing were satisfied. Since the variables were normally distributed, parametric tests were applied to the data. Descriptive statistics were expressed as frequencies and percentages for categorical variables. The distribution of the horizontal position of the mental foramen among the three skeletal groups was compared using the Fisher-Freeman-Halton exact test. As each participant contributed bilateral mental foramen measurements, right and left sides were analysed separately wherever applicable. Side-specific analyses were performed to account for potential within-subject correlation and to facilitate comparison of skeletal groups on each side independently. Differences in the distance from the mental foramen to the adjacent root apex and the distance from the superior border of the mental foramen to the inferior border of the mandible among the three skeletal groups were analysed using one-way ANOVA. Side-wise comparisons of right and left distance measurements within each skeletal group were performed using the paired *t*-test. The relationship between age and continuous distance variables was analyzed using Pearson's correlation coefficient,whereas differences in continuous distance variables between sexes were evaluated using the independent-samples *t*-test. Intra-examiner reliability for linear measurements was assessed using the intraclass correlation coefficient (ICC) based on a two-way mixed-effects model with single measurements and absolute agreement [ICC (3,1)]. Measurement error was additionally quantified using Dahlberg's formula. Intra-examiner agreement for ordinal measurements was assessed using weighted kappa. Given the exploratory nature of this observational study and the relatively small sample size, no formal adjustment for multiple comparisons was applied. Therefore, *p*-values should be interpreted as exploratory rather than confirmatory. A two-sided *p*-value <0.05 was considered statistically significant for all analyses.

## 3 Results

The mean age of the participants was 23.08 ± 2.75 years, 22.54 ± 4.39 years and 21.77 ± 3.06 years in the study groups—prognathic, retrognathic and orthognathic mandibles. In the prognathic and orthognathic groups, 53.8% were males and 46.2% were females, while the retrognathic group comprised of 46.2% males and 53.8% females. In orthognathic mandibles, P3 (69.2%) was the most common position on the right and left sides. The predominant position in the retrognathic mandible was P5–46.2% (right) and 38.5% (left). In the prognathic mandible, P4 was the most common position—53.8% (right), while P3 (46.2%) predominated in the left. Significant differences were seen in the position of the MF between the groups only on the right side (Table 1) while there was no significance in the left side (*p* = 0.124).

**Table 1:** Association between horizontal position of the mental foramen on the right side with mandibular sagittal skeletal pattern.

Variable		Groups			Total	Test	*p* value
		Prognathic mandible N (%)	Retrognathic mandible N (%)	Orthognathic mandible N (%)	N (%)	Statistic^a^	
Mental foramen (right)	P2	1 (7.7)	0 (0.0)	0 (0.0)	1 (2.6)	13.959	0.012*
	P3	3 (23.1)	5 (38.5)	9 (69.2)	17 (43.6)		
	P4	7 (53.8)	2 (15.4)	4 (30.8)	13 (33.3)		
	P5	2 (15.4)	6 (46.2)	0 (0.0)	8 (20.5)		

a Fisher–Freeman–Halton exact test.

* *p* < 0.05 statistically significant.

The distance between the mental foramen and the adjacent root apex showed only minor variations among the prognathic, retrognathic, and orthognathic groups on both sides. On the right side, mean values were 2.84, 3.22, and 2.29 mm, respectively (*p* = 0.474), while on the left side they were 2.97, 2.43, and 2.43 mm, respectively (*p* = 0.669), with no statistically significant differences observed. However, a significant right—left difference was observed specifically only within the retrognathic group (Table 2).

**Table 2:** Comparison of the distance from the mental foramen to the adjacent root apex between the right and left sides in retrognathic mandibular group .

Retrognathic Mandible	Mean (mm)	*N*	SD	95% Confidence Interval of the Difference		*t*	*P* value
				Lower	Upper		
Right	3.222	13	2.389	.004	1.570	2.190	0.049*
Left	2.435	13	1.892				

SD, Standard Deviation.

* *p* < 0.05 statistically significant.

Also, the distance from the superior border of the MF to the inferior mandibular border did not differ significantly among the study groups (Table 3). A statistically significant difference between the sexes was observed (Table 4). The ICC values demonstrated excellent reliability for the parameters across prognathic, retrognathic, and orthognathic groups, with ICCs ranging from 0.948 to 0.996 (*p* < 0.001) (Table 5). Dahlberg's error ranged from 0.156 mm to 0.523 mm. Most variables exhibited low measurement error (<0.4 mm), indicating high reproducibility of the CBCT measurements. The largest measurement error was observed for the retrognathic left distance from the superior border of the mental foramen to the inferior border of the mandible (0.523 mm), although reliability remained excellent (ICC = 0.948) (Table 5). Intra-observer agreement for ordinal variables was assessed using weighted Cohen's kappa. Perfect agreement was observed between the two assessments (weighted *κ* = 1.00) for all the observations. Confidence intervals were not estimated because complete agreement was observed between the two repeated assessments.

**Table 3:** Comparison of the distance from superior border of mental foramen to the inferior border of mandible among mandibular sagittal skeletal patterns.

Study groups		*N*	Mean (mm)	SD	95% Confidence Interval for Mean		*F*	*P* value
					Lower Bound	Upper Bound		
Right	Prognathic	13	12.729	1.828	11.625	13.834	0.942	0.399
	Retrognathic	13	12.515	2.466	11.025	14.006		
	Orthognathic	13	11.650	2.025	10.426	12.874		
Left	Prognathic	13	12.932	1.969	11.742	14.121	0.917	0.409
	Retrognathic	13	11.995	2.449	10.515	13.475		
	Orthognathic	13	11.971	1.701	10.943	12.998		

SD, Standard Deviation.

**Table 4:** Association between sex and the linear distances of the mental foramen from the defined mandibular reference points.

Variable	Groups	*N*	Mean (mm)	SD	*t*	*P* value
Adjacent root apex (right) to MF	Male	20	2.421	2.135	−1.234	0.225
	Female	19	3.170	1.607		
Adjacent root apex (left) to MF	Male	20	2.315	1.857	−1.107	0.276
	Female	19	2.931	1.601		
Superior border of MF to inferior border of mandible (right)	Male	20	13.186	1.952	2.941	0.006*
	Female	19	11.364	1.916		
Superior border of MF to inferior border of mandible (left)	Male	20	13.297	1.984	3.549	0.001*
	Female	19	11.248	1.587		

MF, Mental foramen.

* *p* < 0.05 statistically significant.

**Table 5:** Intra examiner variability.

Variable	ICC	95% CI	*P* value	Dahlberg Error (mm)	Interpretation
Prognathic—Right MF to adjacent root apex	0.985	0.945–0.995	<0.001	0.218	Excellent reliability; low measurement error
Prognathic—Left MF to adjacent root apex	0.977	0.930–0.993	<0.001	0.259	Excellent reliability; low measurement error
Prognathic—Right superior border of MF to inferior border of mandible	0.976	0.904–0.997	<0.001	0.276	Excellent reliability; low measurement error
Prognathic—Left superior border of MF to inferior border of mandible	0.973	0.913–0.992	<0.001	0.299	Excellent reliability; low measurement error
Retrognathic—Right MF to adjacent root apex	0.996	0.987–0.999	<0.001	0.156	Excellent reliability; very low measurement error
Retrognathic—Left MF to adjacent root apex	0.992	0.9736–0.997	<0.001	0.272	Excellent reliability; low measurement error
Retrognathic—Right superior border of MF to inferior border of mandible	0.982	0.942–0.994	<0.001	0.329	Excellent reliability; low measurement error
Retrognathic—Left superior border of MF to inferior border of mandible	0.948	0.840–0.984	<0.001	0.523	Excellent reliability; moderate measurement error
Orthognathic right- Right MF to adjacent root apex	0.953	0.854–0.985	<0.001	0.296	Excellent reliability; low measurement error
Orthognathic—Left MF to adjacent root apex	0.960	0.874–0.988	<0.001	0.306	Excellent reliability; low measurement error
Orthognathic—Right superior border of MF to inferior border of mandible	0.977	0.930–0.993	<0.001	0.291	Excellent reliability; low measurement error
Orthognathic—Left superior border of MF to inferior border of mandible	0.964	0.890–0.989	<0.001	0.315	Excellent reliability; low measurement error

MF, Mental foramen; ICC, Intraclass correlation coefficient; CI, Confidence Interval.

## 4 Discussion

The present CBCT-based study evaluated the position of the mental foramen in relation to sagittal skeletal patterns of the mandible, such as prognathic, retrognathic, and orthognathic mandibles.

The most common horizontal position of the MF in our study, regardless of the mandibular morphology, was P3 followed by P4. While our findings are in agreement with studies conducted in Saudi and Brazilian populations (8, 9), variations in the predominant horizontal position of the mental foramen have been reported across different ethnic groups, with P4–P3 and P4–P5 positions documented in Lebanese, Pakistani, Peruvian and Indian (7, 10–12). A systematic review conducted by Barbosa et al. in populations across Asia, Europe, Africa, North and South America, reported that the most prevalent position was in line with the second premolar followed by a position between the premolars, corresponding to the P4 and P3 classification used in our study (13). In a systematic review conducted by Pele et al, the mental foramen was located between the two premolars or apically to the second premolar, corresponding to P3 and P4 respectively (14), although they did not stratify geographical distribution. Ngeow et al, in their comprehensive review reported that Western populations generally demonstrated the mental foramen between the first and second premolars (P3), whereas Asian populations more commonly showed its position in line with or closer to the second premolar (P4) (15). These differences further support the influence of ethnic and population-related factors on the anatomical position of the mental foramen (10). However, unlike previous studies that primarily assessed mental foramen position without skeletal pattern classification, the present study evaluated variations in relation to sagittal mandibular morphology using CBCT. When stratified by sagittal skeletal pattern, differences in the distribution of the mental foramen were observed. Orthognathic mandibles revealed a bilateral P3 position, whereas P5 was prevalent in retrognathic mandibles. Prognathic mandibles exhibited an asymmetric pattern of distribution with P4 on the right and P3 on the left, respectively. Statistically significant differences in the horizontal position of the MF were seen only on the right side and not on the left.The clinical relevance of mental foramen localization is significant in procedures such as mental nerve block anesthesia and anterior mandibular surgeries. It is recommended that the needle insertion is posterior to the second premolar with anterior angulation so that the anesthetic is deposited in the vicinity of the nerve and decreasing the probability of nerve damage (13). The present findings highlight the importance of preoperative radiography for accurate localization of the mental foramen to minimize the risk of iatrogenic nerve injuries.

The distance between the mental foramen and adjacent root apex did not show statistically significant differences among sagittal skeletal patterns, except in retrognathic mandibles, where a significant difference was observed between the right and left sides. Similar measurements were reported by Al-Mahalawy et al. and Aldosimani et al., who highlighted the proximity of the mental foramen to the mandibular premolar region using CBCT, although they did not evaluate skeletal pattern-based differences (16, 17).

Statistically significant sex-based differences were observed in the distance from the superior border of the mental foramen to the inferior border of the mandible on both sides. Similar findings have been reported in previous studies (18). Chandra et al. (18), however, assessed this parameter using panoramic radiographs, which are inherently prone to distortion and superimposition when compared to CBCT imaging. In contrast, Fontenele et al. (9) did not observe sex-based differences in mental foramen position, which may be attributed to differences in population characteristics or study methodology. The significantly greater distance between the mental foramen and the inferior border of the mandible observed in males may partly reflect overall sexual dimorphism in mandibular dimensions rather than a true positional variation of the mental foramen itself. Previous radiographic and morphometric studies have established greater mandibular dimensions in males in both Indian and non-Indian populations (19–21). The CBCT-based evaluation of mental foramen position in relation to sagittal skeletal patterns represents a key strength of this study and contributes preliminary anatomical reference data that may assist clinicians during mandibular surgical planning.

### 4.1 Limitations

Limitations of this study include the moderate sample size drawn from a relatively limited geographic region, which may limit generalizability. Additionally, the study was restricted to young adults aged 18–30 years, and developmental or age-related variations in mental foramen position were not assessed. The observed sex-related differences may partly reflect general mandibular sexual dimorphism rather than an isolated variation in mental foramen position. The measured distances may have been influenced by variations in tooth position, root inclination, and overall mandibular dimensions, factors that were not specifically evaluated in the present study. Future studies with larger, multi-centre cohorts could further validate these findings for broader clinical applicability and evaluate anatomical variations like accessory mental foramina, bifid mental canals, and anterior loops of the inferior alveolar nerve due to their potential clinical significance.

## 5 Conclusion

CBCT provides a distinct advantage over panoramic imaging, providing three-dimensional visualization and precise morphometric assessment. In the present study, significant differences in the horizontal position of the mental foramen among skeletal patterns were observed on the right side, whereas no significant differences were seen in the left side. In the retrognathic mandible group, the distance between mental foramen and adjacent root apex demonstrated a significant right-left difference. These findings partially support the proposed hypothesis and indicate that CBCT may aid in the preoperative localization of the mental foramen for mandibular surgical planning.

## Data Availability

The raw data supporting the conclusions of this article will be made available by the corresponding author on request.
